# Carcinoma associated fibroblasts (CAFs) promote breast cancer motility by suppressing mammalian Diaphanous-related formin-2 (mDia2)

**DOI:** 10.1371/journal.pone.0195278

**Published:** 2018-03-29

**Authors:** Kaitlyn M. Dvorak, Krista M. Pettee, Kaitlin Rubinic-Minotti, Robin Su, Andrea Nestor-Kalinoski, Kathryn M. Eisenmann

**Affiliations:** 1 Department of Cancer Biology, University of Toledo Health Science Campus, Toledo, Ohio, United States of America; 2 Department of Surgery, University of Toledo Health Science Campus, Toledo, Ohio, United States of America; King Faisal Specialist Hospital and Research Center, SAUDI ARABIA

## Abstract

The tumor microenvironment (TME) promotes tumor cell invasion and metastasis. An important step in the shift to a pro-cancerous microenvironment is the transformation of normal stromal fibroblasts to carcinoma-associated fibroblasts (CAFs). CAFs are present in a majority of solid tumors and can directly promote tumor cell motility via cytokine, chemokine and growth factor secretion into the TME. The exact effects that the TME has upon cytoskeletal regulation in motile tumor cells remain enigmatic. The conserved formin family of cytoskeleton regulating proteins plays an essential role in the assembly and/or bundling of unbranched actin filaments. Mammalian Diaphanous-related formin 2 (mDia2/DIAPH3/Drf3/Dia) assembles a dynamic F-actin cytoskeleton that underlies tumor cell migration and invasion. We therefore sought to understand whether CAF-derived chemokines impact breast tumor cell motility through modification of the formin-assembled F-actin cytoskeleton. In MDA-MB-231 cells, conditioned media (CM) from WS19T CAFs, a human breast tumor-adjacent CAF line, significantly and robustly increased wound closure and invasion relative to normal human mammary fibroblast (HMF)-CM. WS19T-CM also promoted proteasome-mediated mDia2 degradation in MDA-MB-231 cells relative to control HMF-CM and WS21T CAF-CM, a breast CAF cell line that failed to promote robust MDA-MB-231 migration. Cytokine array analysis of CM identified up-regulated secreted factors in WS19T relative to control WS21T CM. We identified CXCL12 as a CM factor influencing loss of mDia2 protein while increasing MDA-MB-231 cell migration. Our data suggest a mechanism whereby CAFs promote tumor cell migration and invasion through CXCL12 secretion to regulate the mDia2-directed cytoskeleton in breast tumor cells.

## Introduction

Approximately 90% of cancer-related deaths are due to advanced metastatic disease [1]. In metastatic breast cancer, invasive primary tumor cells can migrate to regional lymph nodes en route to frequently colonized secondary sites such as bone, liver, brain, lungs, and other tissues. During metastatic dissemination, tumor cells take cues from their local environment. The tumor microenvironment (TME) is a heterogeneous and diverse population of cells surrounding tumors. It is comprised of stromal cells (*i*.*e*., endothelial cells, tumor associated macrophages (TAMs)), carcinoma-associated fibroblasts (CAFs), individual tumor cells, and extracellular matrix (ECM) proteins [2]. In breast cancer, connective tissue and glandular structures are transformed into this cancer-promoting microenvironment. Specifically, CAFs have been implicated in cancer progression due to their innate ability to secrete pro-tumorigenic factors into the TME. In particular, cytokines and chemokines increase cancer cell proliferation, migration/invasion, metastasis, and therapeutic resistance [3–6]. For instance, TGF-β has a well-established role in fibroblast activation and subsequent conversion to a CAF phenotype. In a breast cancer model, primary macrophages increased transendothelial migration of primary breast cancer xenografts mediated by colony-stimulating factor-1 (CSF-1) and increased expression of CSF-1 receptor on tumor cells [7]. In order to target this pro-tumorigenic region and inhibit TME:tumor cell reciprocal signaling, we must first identify proteins underlying TME-driven tumor cell invasion.

During metastatic dissemination, tumor cells must first escape the primary tumor and invade through the adjacent ECM and TME [8–10]. Tumor cells possess a dynamic cytoskeleton which allows them to shift between different motility programs and regulate actin-rich protrusive structures that promote cell motility in response to environmental cues within the TME [11]. For instance, soluble factors from breast cancer cells upregulated the oncoprotein YAP in adjacent fibroblasts which in turn increased actin cytoskeleton contractility and created a self-sustaining, activated CAF phenotype with enhanced secretion of pro-tumorigenic factors [12]. In a pancreatic cancer model, CAFs expressing high levels of the cytoskeletal protein palladin showed increased Cdc42-dependent matrix remodeling that promoted invadopodia formation [13].

The microtubule and actin cytoskeleton are regulated, in part, by the conserved mammalian diaphanous-related (mDia1-3) formin [14] family of cytoskeleton proteins. This family of autoregulated proteins polymerizes, and, in some cases, bundles linear filamentous actin (F-actin), and stabilizes microtubules [15–17]. As mDia formins nucleate and polymerize actin, force is generated by these newly synthesized filaments and this force deforms the cell membrane, creating protrusive structures underlying cell migration and invasion [18]. These structures include spike-like filopodia, broad sheet-like lamellipodia [19], membrane ruffles [19], invadopodia [20, 21], and non-apoptotic membrane blebs [22–24].

mDia formins are regulated through autoinhibition. Autoinhibited mDia proteins exist in a closed or inactive confirmation by an interaction between the Dia inhibitory domain (DID) and the Dia-autoregulatory domain (DAD), which inhibits the functional FH2 domain from associating with actin [25]. Autoinhibition is released when a GTP-bound Rho-GTPase binds to the GTPase binding domain (GBD), thereby disrupting the DID-DAD interaction and releasing the protein into an open, functional confirmation [26, 27]. In addition to cell motility, formins also play a role in cytokinesis, vesicular transport, and transcriptional regulation [28–32]. Formin dysregulation can therefore underlie various pathologies. *DIAPH1* (encoding mDia1) knockout mice had reduced T cells in the peripheral lymphoid organs and T cell:ECM adhesion and migration were inhibited [33, 34]. Loss of mDia1 also impacts other immune cells. *DIAPH1* knockout, in conjunction with *WASP* knockout resulted in defective neutrophil polarization and chemotaxis [35, 36]. Loss of mDia1 expression and function was shown to underlie myeloproliferative and myelodysplastic syndromes [37].

mDia formins were identified as potential therapeutic targets to block tumor cell motility and invasion. Indeed, mDia1 functions in a feedback loop to stimulate mDia1, LARG, RhoA signaling, which in turn modulates cancer cell morphology and invasion [38]. mDia1 was shown to be important for lamellae and filopodia formation following EGF stimulation in MTln3 breast adenocarcinoma cells [39]. mDia1-3 were shown to be important for invadopodia formation and subsequent matrix degradation [40]. mDia2, which is encoded by *DIAPH3*, increased invasive cell egress from epithelial ovarian cancer spheroids [41]. Functional inhibition of mDia2 through association with its negative regulator, Dia-interacting protein (DIP), caused non-apoptotic blebbing, a hallmark of amoeboid motility in breast tumor cells [42]. Conversely, mDia2 activation using small molecule agonists inhibited glioblastoma invasion and migration both *in vitro* and *ex vivo* [43]. Thus, the role of mDia proteins within different tumor microenvironments is likely complex and dictated by specific environmental cues.

In this study, we sought to understand how CAF-soluble factors affect the mDia-directed F-actin cytoskeleton in MDA-MB-231 human breast adenocarcinoma cells. Here we demonstrated conditioned media (CM) from WS19T breast tumor-adjacent CAFs significantly increases MDA-MB-231 breast tumor cell migration and invasion, and is correlated with significant loss of mDia2 protein expression through a proteasomal-dependent mechanism. *DIAPH3* expression was not diminished in response to CAF-CM treatment. Finally, we determined by membrane-based cytokine array that stromal-secreted CXCL12 is a significantly upregulated component of CAF-CM that underlies mDia2 loss in MDA-MB-231 cells and the resultant increase in cell migration.

## Methods and materials

### Cell lines, chemicals, and reagents

MDA-MB-231 breast cancer cells were from ATCC (CRM-HTB-26). Human mammary fibroblasts (HMF) were a kind gift from Dr. Saori Furuta (University of Toledo, Toledo, OH and originally acquired from ScienCell Research Laboratories). WS19T and WS21T human breast carcinoma-associated fibroblasts were kind gifts from Dr. Julie Boerner (Karmanos Cancer Institute, Detroit, MI) [44], and NIH 3T3 fibroblasts were kind gifts from Dr. Kandace Williams (University of Toledo, Toledo, OH) and were originally acquired from ATCC (CRL-1658). MCF10A (CRL-10317) and MCF7 (HTB-22) cells were purchased from ATCC. MDA-MB-231 cells, WS19T and WS21T fibroblasts, and NIH-3T3 fibroblasts were maintained in DMEM (Hyclone) containing 10% FBS (vol/vol), 100 U/ml penicillin, and 100 μg/ml streptomycin. HMFs were maintained in ScienCell fibroblast media, 2% FBS, 1% penicillin/streptomycin solution, and 1% of fibroblast growth supplement [45, 46]. Cells were incubated at 37°C with 5% CO_2_ in a humidified environment.

Anti-mDia2, -mDia1, -ROCK1, -β-catenin, and -GAPDH polyclonal rabbit antibodies were from Proteintech and were used at 1:200 dilutions for immunofluorescence and 1:2,000 for western blotting. Anti-RhoA mouse monoclonal antibodies were from Cytoskeleton (1:1,000 dilution). Anti-tubulin rabbit polyclonal antibodies were from Abcam (1:10,000 dilution). Alexa-Fluor secondary goat anti-rabbit conjugated antibodies were from Invitrogen and were used at 1:200 for immunofluorescence.

Lactacystin (Santa Cruz Biotechnology) was used at 10 μM in dH_2_O with 16h treatment. Vehicle treatments were equal volumes. SMIFH2 in DMSO (EMD Biochemicals; Tocris Bioscience, Avonmouth) was used at 10–40 μM with 16h for wound closure assays and 8-72h for western blot analysis. Cycloheximide in dH_2_O was obtained from Sigma-Aldrich and used at 10 μg/ml for 1-24h. CXCL12/SDF-1α in dH_2_O (R&D Systems) was used at 15–100 ng/ml for 16h for wound closure assays and 8-72h for western blot analysis.

siRNA transfections were performed as previously described [47].

### Western blotting

Whole cell lysates were collected with lysis buffer (0.5M Tris-HCl, pH 6.8, glycerol, 10% SDS (wt/vol), 0.1% bromophenol blue (wt/vol) supplemented with 0.1M diothiothreitol (DTT)) and SDS-PAGE was performed to resolve proteins. Proteins were transferred to PVDF membranes using a BioRad Trans-Blot turbo transfer system. Western blots were exposed using Clarity Western ECL (BioRad) and Alpha Innotech imaging system (Azure Biosystems). Densitometry was performed using ImageJ software.

### Conditioned media preparation

Human mammary, NIH-3T3, WS19T, and WS21T fibroblasts were plated in DMEM growth media, in T-75 culture flasks, and grown to confluence. Media were removed 5 days post-confluence, centrifuged at 1000 rpm to remove cellular debris, and stored at -20°C until use.

### Wound healing assays

Confluent MDA-MB-231 breast cancer cells in 6-well culture plates were scratched with a sterile pipette tip, followed by 16h incubation at 37°C with 5% CO_2_. Immediately following scratching, media were changed to either DMEM, HMF-CM, WS19T-CM or WS21T-CM. Image acquisition occurred at introduction of the scratch (0h) and 16h post scratching. “Wounds” were measured using MetaMorph Image Analysis software to determine percent wound closure. Each condition was performed in triplicate within a single experiment and with a minimum of three experimental repeats.

### Spheroid formation

Spheroids were generated using centrifugation and poly-HEMA coated, low attachment plates as previously described [48]. Coated wells in a 96-well culture plate were seeded with 4,000 cells suspended in DMEM with 10% FBS supplemented with 2.5% of 15μl/ml matrigel (BD Biosciences). Cells were pelleted at 1,000xg for 2m. Spheroids were grown for 72h prior to the start of all assays.

### Spheroid invasion assay

Collagen-1 (BD Biosciences) was used at a concentration of 2mg/ml and prepared as previously described (modified from [49]). 8-well chamber slides (LabTek) were coated with a thin layer of the diluted collagen, spheroids were added in 15μl of media and a thin collagen layer was added on top of the initial collagen layer and spheroid. Slides were incubated at 37°C for 45m to allow for polymerization prior to adding media. Serum-free media (SFM), full growth media (DMEM containing 10% FBS), HMF-CM, or WS19T-CM was used per 8-well slide. Spheroids were imaged upon embedding and 24, 48h, and 72h post-embedding. Media were refreshed every 24h. MetaMorph image analysis software was used to determine the area of each spheroid by drawing a region of interest (ROI) that encompassed at least 90% of the invasion edges. Change in area was used as a measure of invasion. A single experiment included measurements from at least 8 wells of each media type and the experiment was repeated three times.

### RNA isolation and quantitative real-time PCR

RNA was isolated using TRIzol (Invitrogen), and cDNA was generated using the Quantitect Reverse Transcription kit (Qiagen) following the manufacturer’s instructions. Quantitative PCR (qPCR) was completed using Radiant SYBR Green Lo-ROX PCR mix (Alkali Scientific) and an Applied Biosystems 7500 PCR system. Analysis was performed on SDS software as previously described [50]. The average Ct values for tested (DIAPH3) and housekeeping genes (GAPDH and cyclophilin B) were calculated from the individual Ct values generated from the PCR reaction. The average Ct values for the experimental housekeeping genes were subtracted from the tested experimental values for the ΔCt experimental. The average Ct values for the control housekeeping genes were subtracted from the control experimental values for the ΔCt control. The ΔCt control was then subtracted from the ΔCt experimental to produce the ΔΔCt. The final step of analysis is calculating the expression fold change (fold change = 2^-ΔΔCt^). Primers for *DIAPH3*, *PPIB* (encoding cyclophilin B), and *GAPDH* were obtained from Integrated DNA Technologies and used at concentrations following the Radiant protocol. *DIAPH3* mRNA levels were normalized to cyclophilin B and GAPDH mRNAs for analysis. Primer sequences were as follows: *PPIB* (5’-CAT CTG CAC TGC CAA GAC TGA-3’ and 5’-TTG CCA AAC ACC ACA TGC TT-3’), *GAPDH* (5’- GCC TCA AGA TCA TCA GCA ATG C-3’ and 5’ CCA CGA TAC CAA AGT TGT CAT GG-3’) and *DIAPH3* (5’-GCG GGA AAA GGA CTT CAG TAT-3’ and 5’-TCT GTC GGC TTC TCT TAA GAC TTC-3’), and were previously validated and confirmed using BLAST [51].

### Proliferation assay

MTT (Biosynth International) cell viability assay was performed following the manufacturer’s specifications. Metabolic activity was assessed at 16, 24, 48, and 72h. Within a single experiment each condition and time point included nine measurements and the experiment was repeated thrice.

### Immunofluorescence (IF)

Sterile coverslips were coated with 10μg/ml of Collagen-1 at 37°C overnight. Cells plated on collagen-coated coverslips were fixed with 4% paraformaldehyde in PBS, permeabilized with 0.1% Triton X-100, and stained with a primary antibody against mDia2 overnight at 4°C. Alexa-Fluor 488 secondary antibody (Invitrogen), Alexa-594 phalloidin (Molecular Probes), and DAPI (Invitrogen) were applied at 37°C for 1.5h.

### Cytokine antibody array

Custom cytokine antibody arrays were from RayBiotech (S1 Table). The assay was performed following the manufacturer’s instructions. Briefly, treated membranes were exposed using the Alpha Innotech chemilluminescence imaging system (Azure Biosystems). Quantification was performed using ImageJ software and analysis was performed with the RayBiotech analysis software. Background measurements were subtracted and values were normalized to the corresponding target incubated with HMF-CM. WS19T and WS21T-CM were screened three times with each target spotted in duplicate per membrane (S2 Table). Control HMF-CM was screened twice with each target spotted in duplicate per membrane.

### Human CXCL12/SDF1-α ELISA

A human CXCL12/SDF1-α ELISA kit was obtained from Sigma-Aldrich. The assay was performed following manufacturer’s instructions. The provided standard was performed in triplicate. HMF-CM, WS19T-CM, and WS21T-CM were screened in three independent CM sample collections, collected as previously described. Each collection included parallel HMF-CM, WS19T-CM, and WS21T-CM sample. HMF-CM samples were in duplicate, while WS19T-CM and WS21T-CM were assessed in triplicate. Assays were read at 450nm absorbance using a SpectraMax plate reader and results were plotted with SigmaPlot software.

### Statistical analysis

One-tail Student’s t-tests were used with a 95% confidence interval with p < 0.05 interpreted as statistically significant. Standard deviations are shown on histograms.

## Results

### WS19T CAF-derived CM drives increases MDA-MB-231 breast tumor cell motility *in vitro*

Given the notion that CAF-secreted factors promote invasion and metastasis, we hypothesized that mammary tumor-derived CAFs may influence tumor cell motility through modifying the actin cytoskeleton. We first assessed if factors present in WS19T CAF-conditioned CM altered tumor cell motility in wound healing assays. WS19T CAFs are a tumor-adjacent patient-derived mammary carcinoma associated fibroblast cell line [44]. To assess the effects of WS19T-CM conditioned for various lengths of time upon MDA-MB-231 cell motility, wound closure assays were performed using WS19T-CM collected 1-5d post confluency. Confluent cell monolayers were “scratched” (T0) and allowed to fill in the “wound” for 16h. MDA-MB-231 cell wound closure increased in a time -dependent manner in CM collected at 3, 4, and 5d post-confluency, with greatest wound closure in 5d CM (S1 Fig). Therefore, 5d WS19T-CM was used for all subsequent experiments.

**Fig 1:**
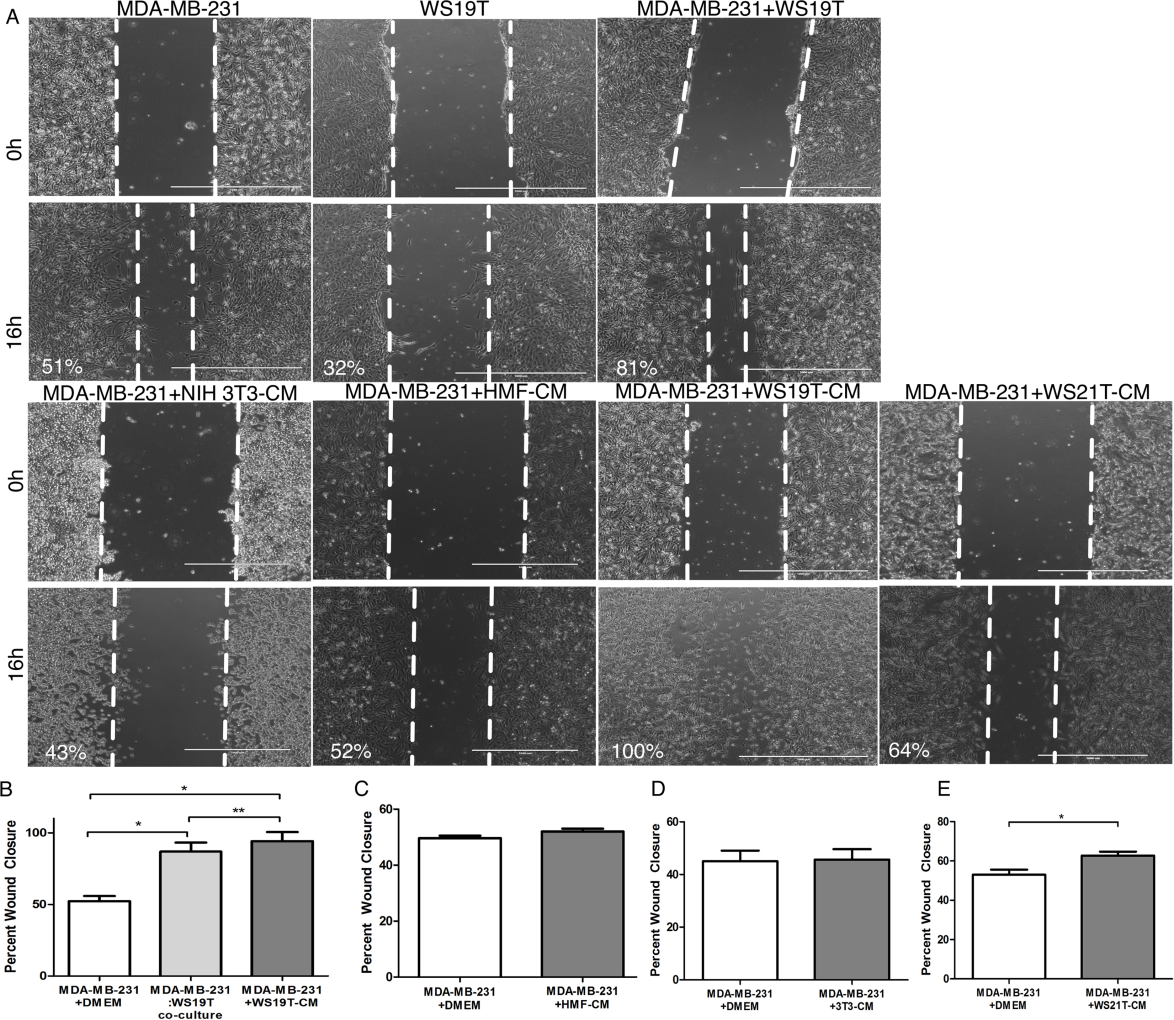
WS19T carcinoma associated fibroblasts (CAFs) and WS19T-CM significantly increase MDA-MB-231 breast adenocarcinoma cell motility. A-E. MDA-MB-231 breast tumor cells, WS19T CAFs, and MDA-MB-231 cells + WS19T CAFs co-culture (1:1 ratio) were plated and grown to confluency. Wound closures were also assessed over 16h in the presence of DMEM growth media, NIH 3T3-CM, HMF-CM, WS19T-CM, or WS21T-CM. For B, *p<0.001; **p<0.03 relative to DMEM control. For E, *p<0.008 relative to DMEM control. Each experiment was performed in triplicate and repeated thrice. Scale bars = 1000μm.

We next assessed the specificity of CAF-CM in MDA-MB-231 cell wound closure assays performed for 16h in the presence of CAF-CM or control CM from normal fibroblasts and additional breast tumor-adjacent CAFs. MDA-MB-231 cells and WS19T CAFs alone had 51% and 32% wound closure, respectively, while co-plating MDA-MB-231 cells and (1:1 ratio) resulted in 81% wound closure (Fig 1A and 1B). However, MDA-MB-231 cells cultured alone in WS19T-CM (conditioned for 5d post-CAF confluency) had 100% wound closure (Fig 1A and 1B). MDA-MB-231 cells incubated in NIH-3T3-CM or HMF-CM, derived from normal human mammary fibroblasts, showed no significant increases in percent wound closure relative to MDA-MB-231 cells wounded in DMEM (Fig 1A, 1C and 1D). CM derived from WS21T fibroblasts (derived from a patient with ER+/PR+ breast cancer [44, 52], modestly increased motility (~64%) in MDA-MB-231 cells compared to control MDA-MB-231 cells in DMEM, yet not as robustly as WS19T-CM (100%) (Fig 1A and 1E).

To assess specificity of CAF-CM upon tumor cell motility, we incubated a panel of normal breast and transformed breast and non-breast tumor cell lines with WS19T-CM. WS19T-CM incubation with MCF-10A and MCF7 cells did not increase MCF-10A motility but did significantly increase MCF-7 motility indicating responsiveness in malignant cells (S2 Fig). Furthermore, when WS19T-CM was applied to the U251 glioblastoma and OVCA429 ovarian cancer cell lines in wound closure assays (S2 Fig), we did not observe significant increases in motility suggesting that breast tumor CAF-secreted factors might be tissue-specific or different concentration requirements drive progression in different tissues of origin.

### WS19T-CM reduces MDA-MB-231 cell proliferation

**Fig 2:**
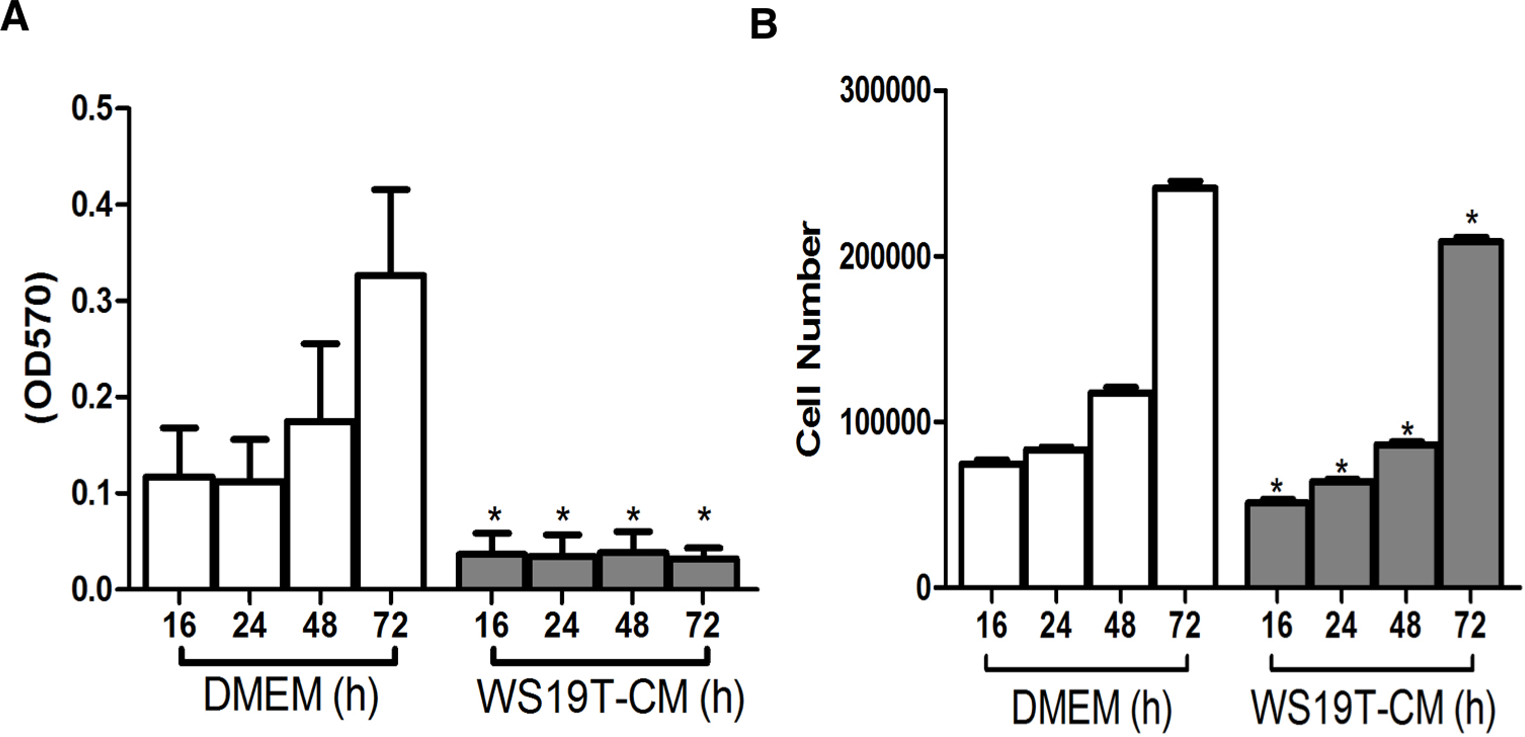
WS19T-CM reduces MDA-MB-231 cell proliferation. A. MTT analysis of MDA-MB-231 cells + DMEM or WS19T-CM for 16-72h. *p<0.0001 relative to DMEM at the corresponding time point. B. 50,000 MDA-MB-231 cells were initially plated into DMEM or WS19T-CM and grown through 72h, manually counting at the indicated time points. *p<0.0001 relative to DMEM control at the corresponding time point. Three independent experiments each consisting of nine replicates per condition.

To validate that increased wound closure throughout 16h was not due to increased cell proliferation, we analyzed the effects of WS19T-CM on MDA-MB-231 proliferation using MTT and cell counting assays. Interestingly, WS19T-CM reduced MDA-MB-231 metabolic activity (Fig 2A) by 55–85% through 72h of WS19T-CM incubation relative to MDA-MB-231 cells in DMEM. We then manually counted cells incubated in DMEM growth media or WS19T-CM. We plated 50,000 cells and assessed growth ever 24h through 72h. MDA-MB-231 cells in the presence of WS19T-CM still proliferated, but did so at a modest yet significantly decreased rate (~2,678 cells/h) compared to the corresponding DMEM-treated cells (~3,125 cells/h) (Fig 2B). Therefore, increased MDA-MB-231 cell proliferation does not account for increased wound closure in response to WS19T-CM.

### WS19T–CM increases MDA-MB-231 cell invasion in a 3D collagen matrix

**Fig 3:**
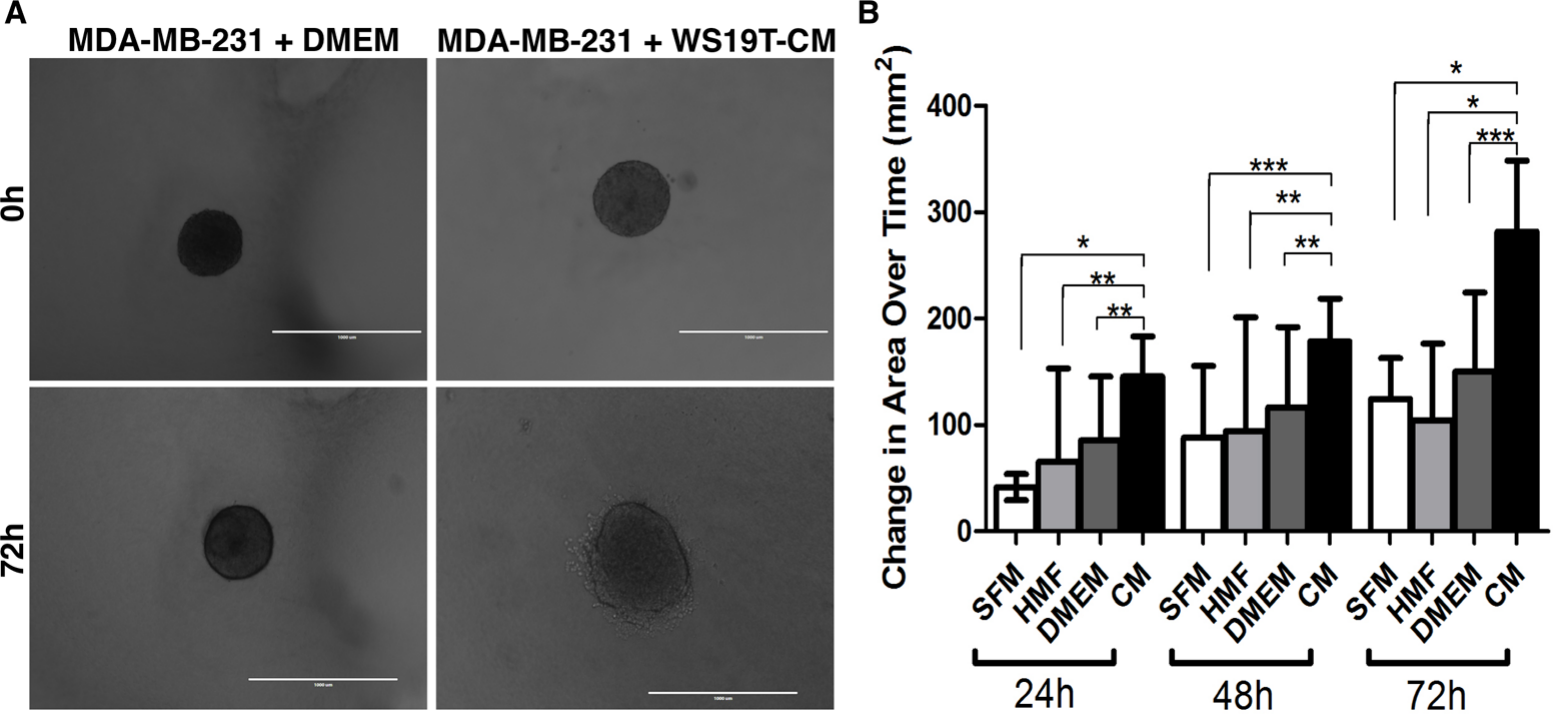
WS19T-CM increases MDA-MB-231 tumor cell invasion. A. Representative images from MDA-MB-231 spheroids embedded in 2 mg/ml Type 1 collagen and incubated with DMEM growth media or WS19T-CM through 72h. Scale bars = 1000 μm. B. MDA-MB-231 spheroids were embedded in collagen and incubated with serum-free media (SFM), human mammary fibroblast-CM (HMF), DMEM + 10% FBS (DMEM), or WS19T-CM (CM). The area of each spheroid was measured at the time of embedding (T0) and at the indicated time points. Data are expressed as change in area relative to T0. Each condition was performed in triplicate and repeated thrice. *p<0.0001; **p<0.03; ***p<0.003.

We next assessed whether WS19T-CM affected MDA-MB-231 cell invasion. MDA-MB-231 spheroids were formed for 72h, embedded in 2mg/ml Type-1 collagen gels, and allowed to invade for an additional 72h [48] (Fig 3A). Embedded spheroids incubated with serum-free medium (SFM), control HMF-derived CM, and DMEM growth media showed moderate invasion through 72h (Fig 3A and 3B). Spheroids embedded with WS19T-CM showed significantly increased invasion compared to controls at corresponding culture times through 72h invasion. Thus, CAF-CM increases MDA-MB-231 motility in both 2D and 3D environments.

### WS19T-CM decreases mDia2 protein expression in MDA-MB-231 cells

**Fig 4:**
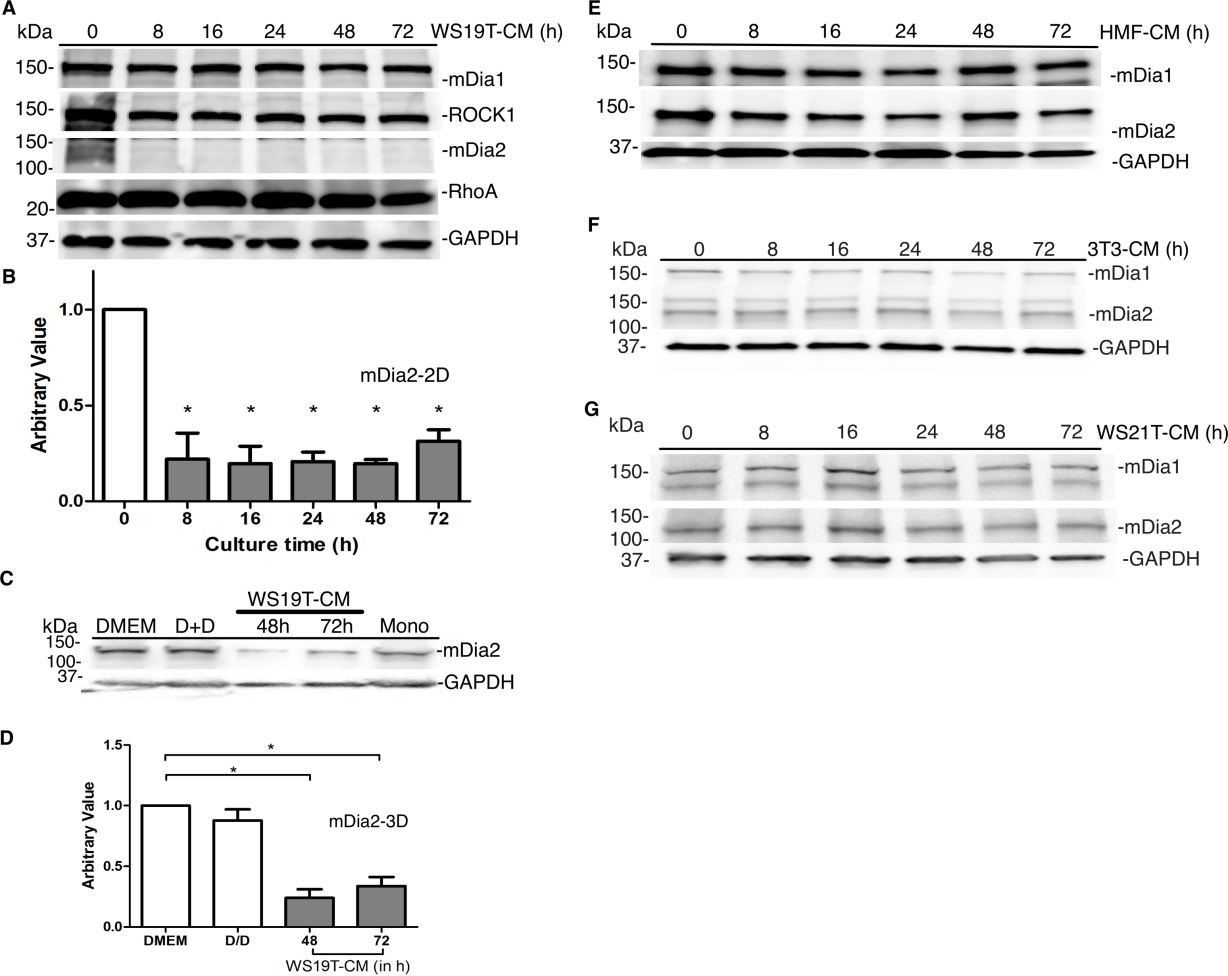
WS19T-CM reduces mDia2 protein expression. A. MDA-MB-231 cells were incubated in monolayers with WS19T-CM for 8-72h and western blotted for the indicated proteins. B. Densitometry was performed on A using Image J and mDia2 expression was normalized to GAPDH and compared to MDA-MB-231 cells in DMEM. *p<0.001 relative to DMEM (0h). C. MDA-MB-231 cells spheroids were cultured in WS19T-CM for 48-72h and cell lysates were blotted for the indicated proteins. Mono = MDA-MB-231 monolayer lysate. The DMEM condition were cells held in DMEM for the duration of the experiment. The D/D condition are cells that were plated in DMEM and underwent a media change at the same time point as the CM condition but was changed back into DMEM. D. Densitometry was performed on C using Image J with mDia2 expression normalized to GAPDH and compared to MDA-MB-231 spheroids in DMEM. *p<0.004 relative to DMEM. Each experiment was repeated thrice. E-G. MDA-MB-231 cells were incubated in monolayers with HMF-CM, 3T3-CM, or WS21T-CM for 8-72h and western blotted for the indicated proteins.

mDia formins assemble non-branched actin filaments that underlie protrusive structures in motile tumor cells [27, 40–43, 53–56]. We next assessed mDia protein expression in 2D in response to CAF-CM, along with RhoA, an upstream activator effecting both mDia and ROCK signaling. In MDA-MB-231 cells incubated in WS19T-CM for 8-72h, mDia2 protein expression significantly decreased within 8h of WS19T-CM treatment relative to MDA-MB-231 cells in DMEM growth media (Fig 4A and 4B) and inhibition was sustained through 72h. RhoA, ROCK and mDia1 protein levels remained relatively unchanged when compared to MDA-MB-231 cells in DMEM (Fig 4A). In 3D, MDA-MB-231 spheroids first formed in DMEM and then cultured in WS19T-CM showed >75% loss of mDia2 expression relative to control spheroids formed in DMEM (D) and/or DMEM-treated (D+D) spheroids (Fig 4C and 4D). Western blot analysis of HMF-CM, NIH 3T3-CM, and WS21T-CM treated MDA-MB-231 cells in 2D cultures showed no detectable mDia2 or mDia1 protein expression changes through 72h relative to MDA-MB-231 cells in DMEM (Fig 4E–4G).

### WS19T-CM mediated mDia2 protein expression loss is recoverable

**Fig 5:**
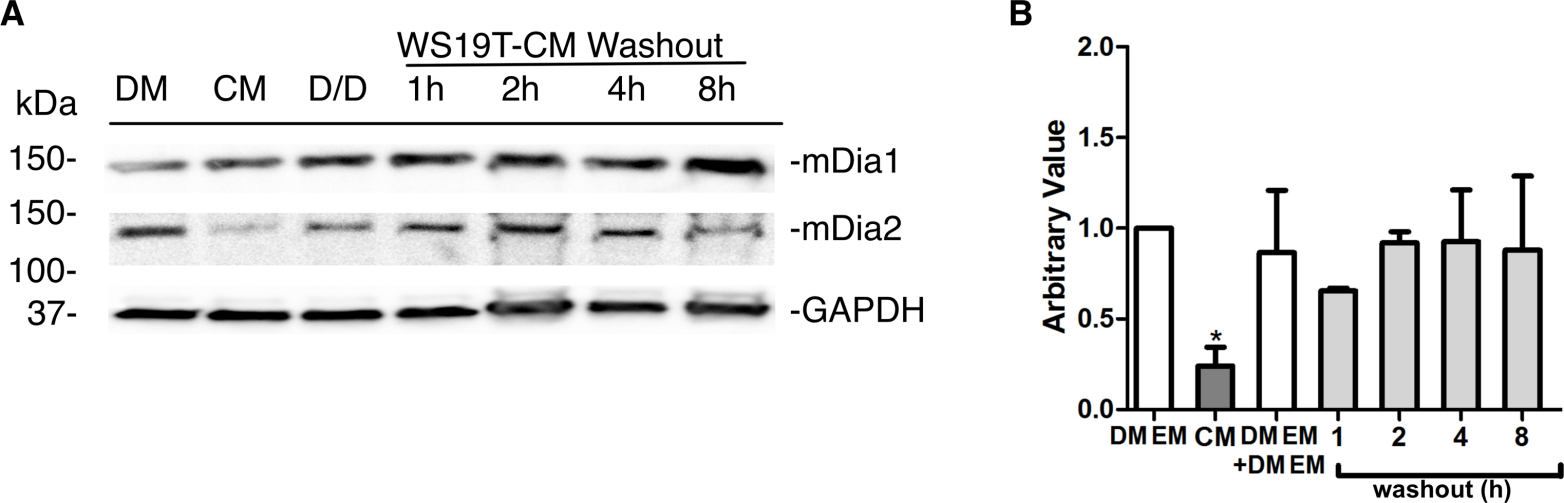
WS19T-mediated mDia2 loss is recoverable upon washout. A. MDA-MB-231 cells were incubated in DMEM with no media change (DMEM), WS19T-CM (CM), or DMEM with a media refresh of DMEM (D/D) for 8h. Cells in WS19T-CM were then released into DMEM after the 8h incubation. Western blots were performed on cell lysates for the indicated proteins. B. Densitometry was performed on A using Image J with mDia2 expression normalized to GAPDH and compared to DMEM. *p<0.03 relative to DMEM. The experiment was performed in quadruplicate.

Kinetic washout assays were performed to evaluate if mDia2 loss in response to WS19T-CM was recoverable (Fig 5). MDA-MB-231 cells were first incubated in either DMEM growth media or WS19T-CM for 8h. The respective media were then washed out and replaced with DMEM growth media. mDia2 protein was re-expressed as early as 1h after the CM-washout (dark grey vs. light grey bars, Fig 5B). Levels approached DMEM control levels (white bars) by 2h post washout. mDia1 protein levels remained relatively unchanged with time (Fig 5A and 5B) in the presence of WS19T-CM and throughout the washout.

### WS19T-CM does not affect mDia2 mRNA levels

**Fig 6:**
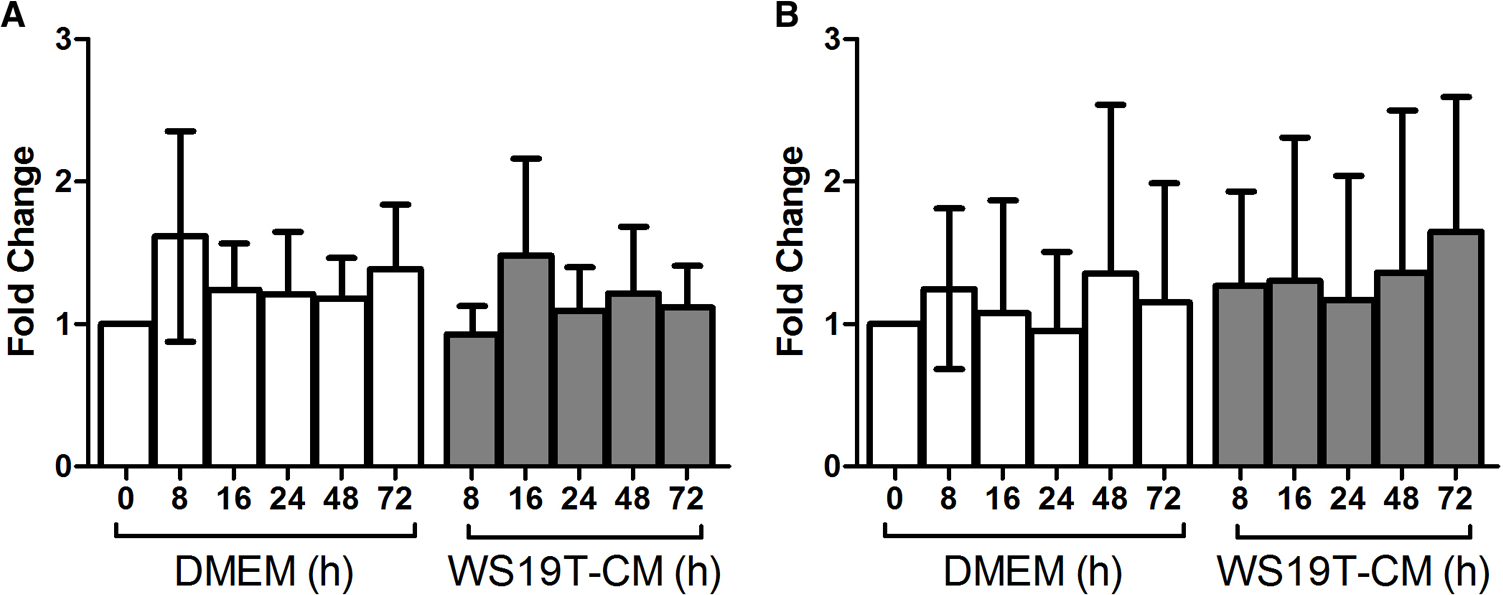
WS19T-CM does not decrease mDia2 mRNA levels. A. MDA-MB-231 cells were treated for the indicated times with either DMEM growth media or WS19T CM, and qRT-PCR performed in triplicate. Data are expressed as expression fold change (2^-ΔΔCt^) relative to A. cyclophilin B *(PPIB)* or B. *GAPDH*. No significant differences were observed. The experiment was performed 4 times.

To determine if mDia2 expression loss is at the level of the mDia2 mRNA, qRT-PCR was performed with primers recognizing *DIAPH3* or control genes encoding cyclophilin B *(PPIB)* or *GAPDH*. MDA-MB-231 control and *DIAPH3* mRNA levels were assessed after 8-72h of WS19T-CM treatment (Fig 6), paralleling mDia2 protein expression (Fig 4). *DIAPH3* levels remained statistically unchanged when normalized to *PPIB* or *GAPDH* mRNA, compared to the corresponding DMEM treatment time points (Fig 6A and 6B). Thus, WS19T-CM regulates mDia2 protein expression and not *DIAPH3* mRNA levels.

### mDia functional inhibition does not affect mDia2 protein levels or cell motility

**Fig 7:**
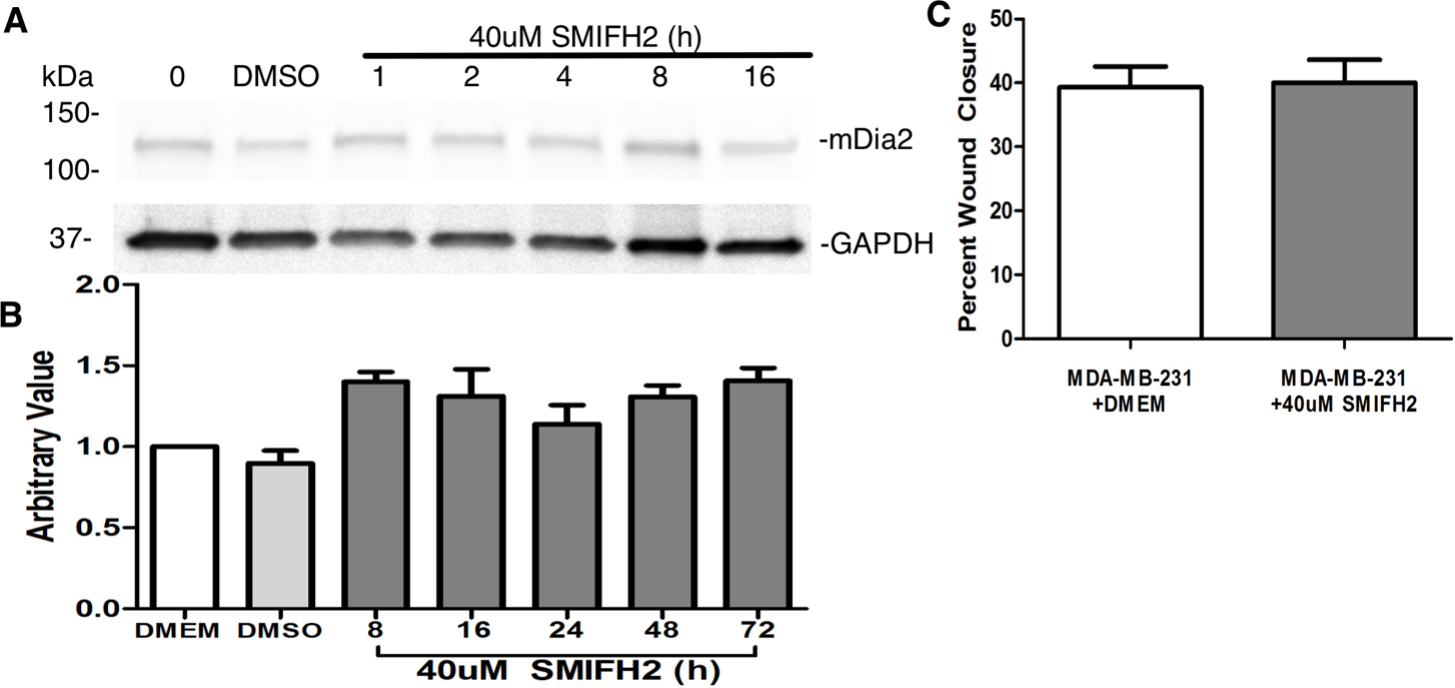
mDia2 functional inhibition does not affect mDia2 protein expression. A. MDA-MB-231 cells were grown to 60% confluency and treated with vehicle (DMSO) or 40μM SMIFH2 for 1-16h prior to lysate collection and Western blotting. B. Densitometry from A. where mDia2 was normalized to GAPDH and compared relative to DMEM. C. MDA-MB-231 cells were treated with 40μM SMIFH2 starting at T0 continuously for 16h during a wound closure experiment. Experiments were repeated thrice.

mDia2 functional inhibition using the small molecule inhibitor of FH2 domain, SMIFH2, resulted in loss of mDia2 protein in U2OS cells within 2-16h, and within 5h in HEK 293T, A375, and MDA-MB-231 cells through an unidentified non-proteasomal mechanism of protein degradation [51]. SMIFH2 functionally inhibits the FH2 domain of mDia formins and prevents F-actin nucleation, decreases formin affinity for the barbed end of F-actin, and reduces F-actin elongation [57]. We assessed whether mDia functional inhibition decreased mDia2 protein expression. We first validated the functionality of the SMIFH2 used in these studies by assessing induction of non-apoptotic plasma membrane blebs in MDA-MB-231 cells, a functional consequence we previously observed [42]. Indeed, 10μM SMIFH2 induced robust blebbing, confirming functionality of individual lots of SMIFH2 (data not shown). MDA-MB-231 cells treated with 40μM SMIFH2 for 8-72h revealed no loss of mDia2 protein expression compared to MDA-MB-231 cells in DMEM and DMSO-treated cells (Fig 7A and 7B). In our system, cell motility was unaffected upon mDia2 functional suppression, with no significant difference in percent wound closure between DMEM- and SMIFH2-treated cells (Fig 7C) after 16h.

### WS19T-CM reduces mDia2 protein expression through a proteasome-dependent mechanism

**Fig 8:**
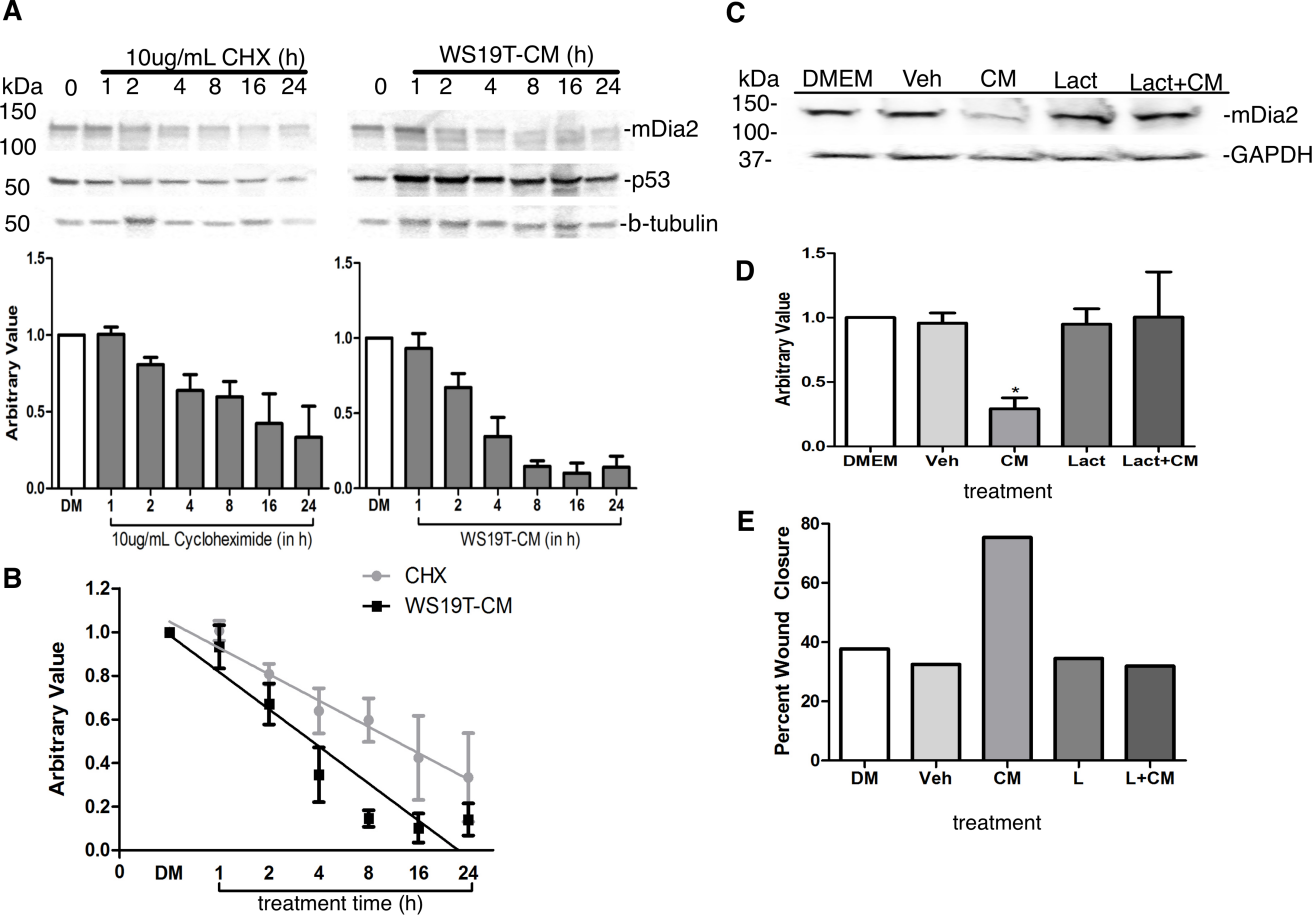
WS19T-CM mediated mDia2 loss is proteasome-dependent. A. MDA-MB-231 cells were treated with 10μg/mL cycloheximide (CHX) or WS19T-CM for 1-24h. Cell lysates assessed by Western blotting. mDia2 expression was normalized to GAPDH of the respective 0h condition and compared to mDia2 expression at the respective 0h condition. B. Normalized mDia2 expression was graphed versus time. A fit line was generated and the resulting equation was used for extrapolating unknown values. All experiments were repeated thrice. C. MDA-MB-231 cells were grown to 60% confluency. Cells were incubated in DMEM, vehicle (water), WS19T-CM (CM), lactacystin (Lact or L), or WS19T-CM+ lactacystin (Lact+CM, L+CM) for 16h prior to cell lysate collection and Western blotting. D. Densitometry was performed on C, where mDia2 expression was normalized to GAPDH expression and compared relative to MDA-MB-231 cells in full DMEM. *p<0.01 compared to DMEM. E. MDA-MB-231 cells were treated as in C. at T0h continuously for 16h during a wound closure experiment.

We next sought to understand mechanisms whereby mDia2 protein expression is lost in MDA-MB-231 cells in response to WS19T-CM. We first utilized cycloheximide to determine the half-life of mDia2 in culture and compare to WS19T-CM-affected mDia2 expression kinetics. MDA-MB-231 cells treated with 10μg/mL of cycloheximide yielded an mDia2 half-life of 5.6h (Fig 8A and 8B). When MDA-MB-231 cells were treated with WS19T-CM for the same time course, mDia2 half-life decreases to 3.9h. This shortened mDia2 half-life in the presence of WS19T-CM supports the notion of a mechanism other than the normal turnover of mDia2 protein is at play in response to WS19T-CM in MDA-MB-231 cells.

mDia2 protein expression during the cell cycle is tightly regulated by ubiquitination and subsequent degradation [28]. mDia2 is expressed in S-and G2/M phase with a significant drop following progression into G0/G1 phase. This marked drop is due to poly-ubiquitination followed by degradation. We next assessed whether proteasomal-mediated degradation of mDia2 is initiated by CAF-CM factors. MDA-MB-231 cells were treated with the proteasome inhibitor lactacystin for 16h in the presence of DMEM or WS19T-CM and mDia2 protein expression was evaluated. Neither vehicle-treated nor proteasome inhibitor-treated MDA-MB-231 cells showed loss of mDia2 protein expression when cultured in DMEM (Fig 8C and 8D). Proteasome inhibition in the presence of WS19T-CM restored mDia2 protein expression to that of DMEM and vehicle control levels. Proteasome inhibition also inhibited CM-mediated motility (via wound closure) to levels comparable to MDA-MB-231 cells cultured in DMEM (Fig 8E).

### WS19T-CM does not affect mDia2 localization

mDia2 protein expression is lost in the presence of WS19T-CM, and is followed by the quick recovery of mDia2 expression upon WS19T-CM washout. To examine if changes in intracellular protein localization underlie mDia2 recovery, we visualized mDia2 localization in MDA-MB-231 cells incubated in DMEM, HMF-CM, NIH-3T3-CM, WS21T-CM, and WS19T-CM. Compared to MDA-MB-231 cells in DMEM, the percentages of nuclear vs. cytoplasmic mDia2 were not changed in response to either HMF-CM, NIH-3T3-CM, and WS19T-CM (S3 Fig). Thus, intracellular sequestration does not appear to be a mechanism impacting mDia2 protein expression and/or recovery in our system.

### WS19T-CM contains up-regulated, cancer-associated cytokines

**Fig 9:**
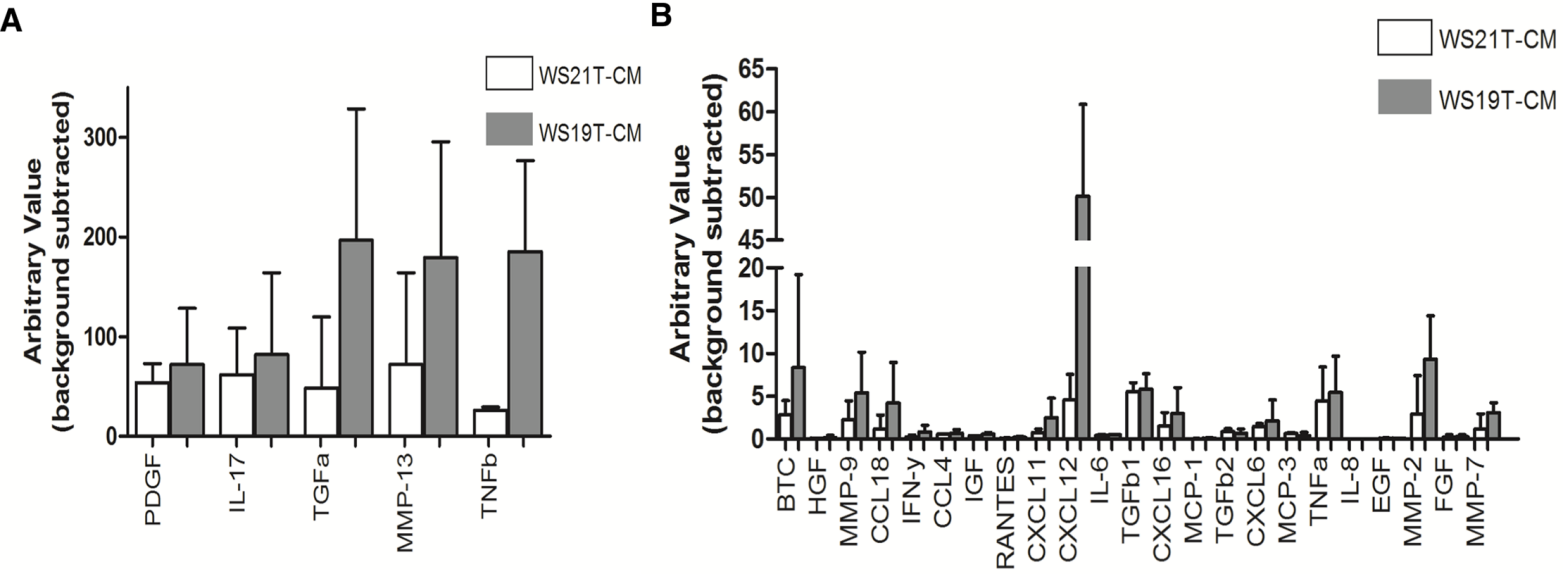
WS19T-CM upregulates a panel of cancer-associated cytokines. A. Conditioned media from HMF, WS21T CAFs, and WS19T CAFs were analyzed using membrane-based cytokine antibody arrays. Samples were assessed in duplicate. The experiment was repeated three times. B. Densitometry of WS19T-CM and WS21T-CM treated cytokine membranes were normalized to HMF-CM.

To characterize factors present in WS19T-CM that underlie enhanced MDA-MB-231 cell motility and/or loss of mDia2 expression, we performed a cytokine array analysis. HMF-CM, WS19T-CM, and WS21T-CM were applied to cytokine arrays assessing 28 target proteins (S1 Table). HMF-CM was used as a control for baseline levels of these targets in a non-cancerous stromal population and as HMF-CM promoted neither MDA-MB-231 migration nor influenced mDia2 expression. Cytokine targets were chosen based on known CAF-secreted factors and tumor-promoting factors. Cytokines of interest were identified by up-regulation in WS19T-CM arrays relative to WS21T-CM arrays to identify factors with potential to differentially affect MDA-MB-231 cell motility and reduce mDia2 protein expression. TGFα, PDGF, IL-17, TNF-β MMP-13, and CXCL12 all showed dramatically increased expression in the WS19T-CM compared to WS21T-CM (Fig 9A and 9B and S2 Table).

Upregulated cytokines were then prioritized based on previous connections to both cancer progression (*i*.*e*., motility, invasion and metastasis) and the F-actin cytoskeleton. CXCL12, PDGF, and TGFα were further identified as our top three prioritized hits. CXCL12 and its receptor CXCR4 were implicated in cancer cell migration, invasion, and metastasis through formation of chemotactic gradients [58–67]. PDGF signaling regulated actin dynamics through various downstream effectors [68–70] (reviewed in [71]). TGFα increased motility in MDA-MB-231 cells and upregulation was correlated with more aggressive cases of gastro-esophageal junction (GEJ) adenocarcinoma [72, 73].

### CXCL12 is a key effector in WS19T-mediated MDA-MB-231 cell motility

**Fig 10:**
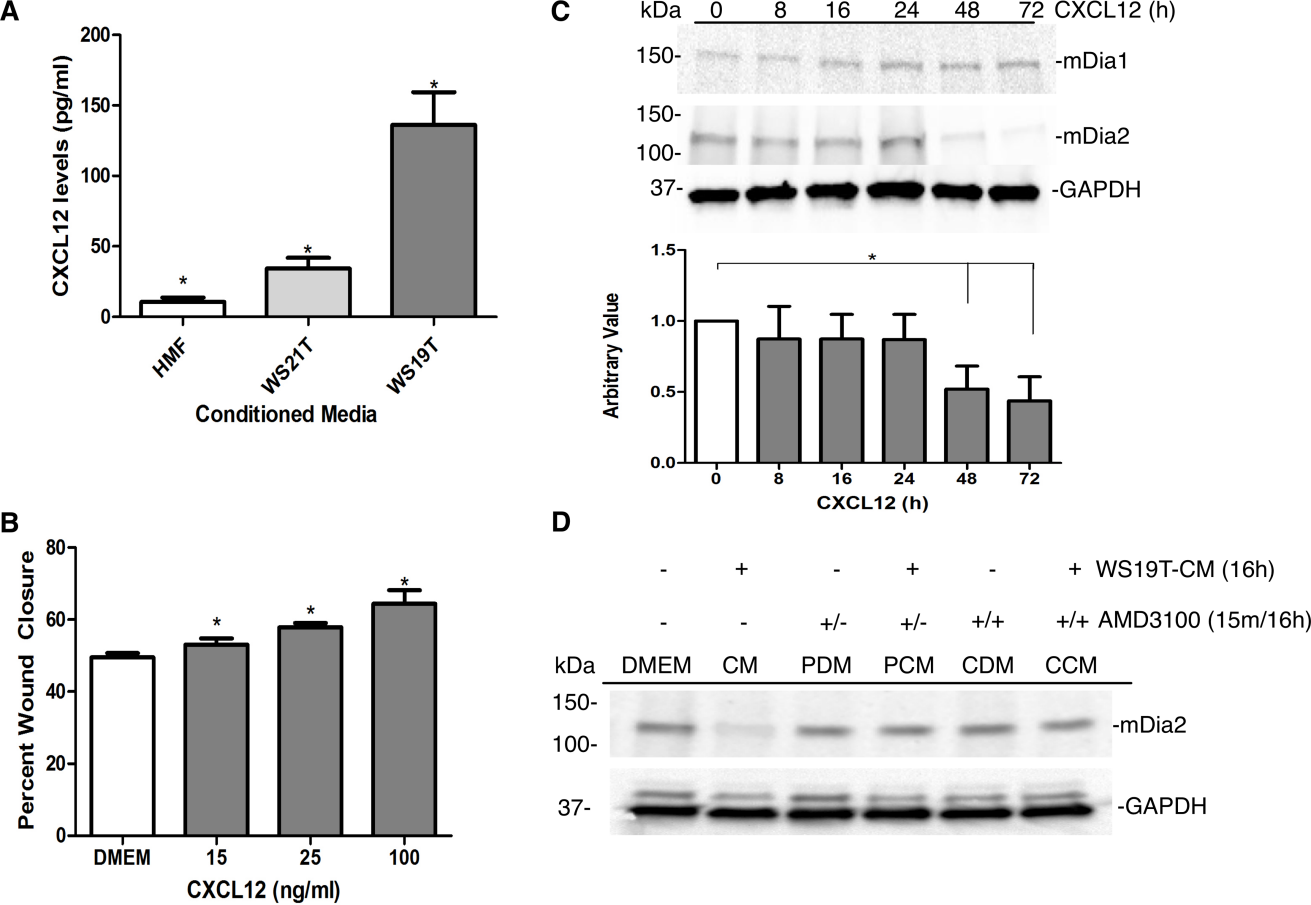
CXCL12 mediates MDA-MB-231 mDia2 downregulation while cell migration. A. HMF, WS21T, and WS19T fibroblasts were plated and CM collected concurrently for each replicate. HMF-CM, WS19T-CM, and WS21T-CM from three independent collections were applied in triplicate to a CXCL12/SDF1α ELISA assay. The experiment was repeated three times. CXCL12 levels were averaged for each CM and compared to HMF-CM. *p<0.0008 HMF-CM relative to WS21T-CM, WS21T-CM relative to WS19T-CM, and HMF-CM relative to WS19T-CM. p<0.001. B. MDA-MB-231 cells were treated with 15, 25, and 100ng/ml CXCL12 and wound closure assays performed for 16h. The assay performed in triplicate and repeated three times. *p<0.001 C. MDA-MB-231 cells were treated with 100ng/ml CXCL12 for 8-72h and cell lysates were Western blotted. mDia2 expression was normalized to GAPDH and compared to the DMEM control. The experiment was repeated three times. *p<0.01 D. MDA-MB-231 cells were treated with WS19T-CM as indicated. Cells were pretreated (P) for 15m with AMD3100, and/or simultaneously and continuously (C) with AMD3100 and CM (CM) or DMEM (DM) for 16h. Cell lysates were Western blotted as indicated.

We initially focused upon CXCL12 and its role in promoting MDA-MB-231 invasion and in regulating mDia2 expression. We first evaluated the physiological levels of CXCL12 within our CM panel via CXCL12/SDF1-α ELISA. We assessed CXCL12 levels in HMF-CM, WS19T-CM, and WS21T-CM. Within HMF-CM, WS21T-CM, and WS19T-CM, CXCL12 levels were measured at ~10.5, 31.0, and 136 pg/mL, respectively (Fig 10A). We therefore observed the highest level of CXCL12 secretion in WS19T-CM—the cell line whose CM most dramatically contributed to migration, invasion, and loss of mDia2 in MDA-MB-231 cells.

To evaluate the effects of CXCL12 upon MDA-MB-231 cell migration, we performed wound closure assays in response to purified CXCL12. We treated MDA-MB-231 cells for 16h with 15, 25, and 100ng/ml CXCL12. CXCL12 treatment resulted in significantly increased wound closure relative to MDA-MB-231 cells in DMEM (~60% closure vs. 45% closure, respectively) (Fig 10B). Modest yet significant increases in wound closure was observed with the lower CXCL12 concentrations as well.

Finally, we evaluated the effects of CXCL12 upon mDia2 expression in MDA-MB-231 cells. MDA-MB-231 cells treated with 100ng/ml CXCL12 for 8-72h had significantly reduced mDia2 protein expression relative to MDA-MB-231 cells in DMEM (Fig 10C). Pre-incubation (PCM) as well as continuous (CCM) treatment of MDA-MB-231 cells with AMD3100, an inhibitor blocking the CXCL12 receptor CXCR4, blocked WS19T-CM-mediated loss of mDia2 protein relative to DMEM growth media-treated cells, supporting a role, in part, for CXCL12 signaling and regulation of mDia2 protein expression in our system (Fig 10D).

## Discussion

In this study, we discovered a role for stromal-derived CXCL12 in breast cancer migration and invasion potentially through proteasome-mediated loss of mDia2 protein expression. WS19T CAF-CM significantly increased motility and invasion of MDA-MB-231 cells relative to MDA-MB-231s in DMEM growth media, or control CM. We observed concomitant and specific loss of mDia2 protein expression with incubation with WS19T CAF-CM. The stromal-derived factor CXCL12 was subsequently identified as a prominent factor enriched in WS19T CAF-CM that promoted, in part, both MDA-MB-231 cell migration, as well as loss of mDia2 protein.

We show a loss of mDia2 expression with increased cell migration and invasion in MDA-MB-231 cells in the presence of WS19T-CM. Indeed, we previously demonstrated that mDia2 expression and/or activity was involved in tumor cell motility. Sustained mDia2 activation using small molecule intramics (IMMs) reduced U87 and U251 glioblastoma invasion [43], while mDia2 functional inhibition promoted amoeboid motility in a MDA-MB-231 breast cancer model [42, 47], and loss of mDia2 function and/or expression increased single cell dissemination in an ovarian cancer spheroid model [41]. Furthermore, *DIAPH3* expression is reduced in invasive prostate cancer [53] and in breast and hepatocarcinoma cells [74]. Thus, tight regulation of mDia2 (and other mDia formins) is an important factor underlying cell motility in a variety of tumor models. Until now, little was known regarding what physiological drivers impact mDia2 expression and/or function. The loss of mDia2 expression in the presence of WS19T-CM highlights a physiologically relevant source of secreted factors targeting and downregulating mDia2, and subsequently promoting cancer cell dissemination and invasion. Interestingly, *DIAPH3* expression was decreased in micro-dissected tumor adjacent stroma derived from invasive breast carcinoma [74–77]. Hence, tight regulation of *DIAPH3*, or mDia2 expression and/function as a mechanism to control cellular transformation and dissemination may not be employed solely by tumor cells, but by the TME cellular constituency as a whole. It must be noted that this is the first tumor cell model of mDia2 regulation in response to CAF-CM. It warrants expanding studies to additional cancer models where a role for mDia2 in tumor invasion and metastasis has been established, such as prostate, hepatocarcinoma and glioblastoma, to test the specificity of this mechanism of mDia2 dependent mode of motility regulation.

We examined the effects of the SMIFH2-mediated mDia suppression in our system to evaluate if mDia functional suppression impacted mDia protein stability and/or expression. Indeed, a previous report correlated SMIFH2-mediated functional suppression with loss of mDia2 expression in select cell lines [51]. In our system, unlike treatment with WS19T-CM, SMIFH2 treatment for 8-72h did not result in loss of mDia2 expression at specific time points, yet it did drive amoeboid conversions and non-apoptotic membrane blebbing (data not shown), as we previously demonstrated [42]. Within a continuous 16h treatment window, however, wound closure was not impacted with SMIFH2 treatment. Cell velocities, persistence, and total distance migrated were not measured, however. These results differ from a previous report, in which MDA-MB-231 cells treated with SMIFH2 lost mDia2 protein within 5h [51]. It is possible that due to the cyclical nature of SMIFH2 action [51], or lot-to-lot variability in the inhibitor, the kinetics of mDia2 expression upon SMIFH2 suppression are subtly altered in our system. Alternatively, functional suppression of mDia2 (via the SMIFH2 mechanism) is not a requisite for mDia2 protein loss. Whether functional suppression of mDia2 precedes protein loss in WS19T-CM-treated MDA-MB-231 is currently under investigation in our lab.

We sought to understand whether the loss of mDia2 was at the level of gene transcription or protein stability/degradation. RT-PCR indicated that levels of *DIAPH3* transcripts are unchanged in the presence of WS19T-CM. We utilized cycloheximide to determine the half-life of mDia2 in culture and compare to WS19T-CM kinetics. The mDia2 half-life in MDA-MB-231 cells treated with CM is significantly shorter than that of cycloheximide-treated cells (~3.9 vs. 5.6h, respectively). Targeting the proteasome with lactacystin both restores mDia2 expression in the presence of CM and blocked the CM-induced increase in cell motility, pointing towards a proteasome-dependent mechanism for loss of mDia2. There is evidence for linkage between mDia2 and the proteasome in tightly regulating mDia2 expression. mDia2, ubiquitin, and the proteasome were shown to directly interact yet mDia2 did not undergo proteasome-mediated degradation in a HEK 293T forced overexpression system [78]. Conversely, mDia2 was expressed through S- and G2/M phase of cell division in HeLa cells and was highly poly-ubiquitinated at the end of M phase, followed by substantial degradation and loss of mDia2 as cells enter G0/G1 phase [28]. Our results support these findings, and moreover, highlight physiological signals triggering mDia2 proteasomal degradation in breast tumor cells.

We noted dramatic upregulation of 6 factors by cytokine array analysis of WS19T-CM, relative to HMF-CM and WS21T-CM. We prioritized factors based upon known established links with cancer cell invasion, metastatic formation, cytoskeleton regulation, or mDia formins. From those criteria, CXCL12, PDGF [69–71], and TGF-α [72, 73] were our top 3 candidates, with initial focus upon CXCL12. We revealed a novel finding that purified CXCL12 promoted mDia2 loss in MDA-MB-231 cells loss through engagement of its receptor CXCR4 (Fig 10), and promoted MDA-MB-231 cell motility. These results indicate an important intersection between CXCL12 signaling and regulation of the mDia2-directed cytoskeleton driving tumor motility.

CXCL12 ELISAs (Fig 10) revealed a CXCL12 average concentration within WS19T CAF-CM of >100 pg/ml. At this concentration, CXCL12 was, in part, sufficient to promote invasion and migration, as well as loss of mDia2 expression. How does this concentration compare to other studies measuring CXCL12 levels in invasive tumors? Serum CXCL12 levels in patients with esophageal cancer were measured at 1.27 ng/ml compared to 0.86 ng/ml in healthy controls [79], while gastric cancer tumors were approximately 3618 ng/ml compared to 1715 ng/ml in the non-cancer control group [80]. Such substantially disparate CXCL12 values could point to distinct functions for CXCL12 in different malignancies and pathologies, as well as additive/synergistic roles for other cytokines in promoting cancer phenotypes. In our studies using purified CXCL12 in monolayer culture, higher concentrations were needed to mimic the effects of WS19T-CM upon MDA-MB-231 migration and mDia2 suppression. Within the context of WS19T CM, CXCL12, likely in conjunction with other enriched cytokines and growth factors, may underlie a more complex system to fully regulate the actin cytoskeleton and cell motility. Within the TME, differences in temporally and spatially local CXCL12 concentrations may differentially influence mDia2 loss and/or increased motility. Collectively, our study does not indicate an exclusive role for CXCL12 in promoting both mDia2 loss and tumor migration. Rather it indicates an important and likely complementary role within a milieu of various cytokines. Future experiments will focus upon the additive or possible synergistic signaling nature of CXCL12 and other cytokines in our system, such as PDGF, in driving mDia2 loss while promoting motility.

In this study, we observed significant increases in breast cancer cell migration and invasion in response to CAF-conditioned media, which was accompanied by dramatic loss of mDia2 expression. We identified CXCL12 as an underlying factor within WS19T-CM that mediates, in part, these phenotypes. Previous work identified a role for exogenous CXCL12 in breast cancer motility and migration, yet the source of CXCL12 influencing tumor motility was uncertain. Here we identify for the first time a physiological source for CXCL12 in the TME- specifically from tumor-adjacent carcinoma associated fibroblasts, and reveal a unique role for the TME in directly influencing tumor cell motility through mDia formin-dependent cytoskeletal regulation. This novel mechanism is a step towards understanding the role of CAF:tumor signaling in cancer progression and identifies potential therapeutic targets that could aid in blocking metastatic dissemination and improving patient prognosis.

## Supporting information

S1 Table List of candidate factors screened by cytokine antibody array. (TIF)

S2 Table Raw data values from cytokine antibody array set. (XLSX)

S1 Fig Extended WS19T-CM culturing increases MDA-MB-231 motility. A, B. WS19T media was conditioned for 1–5 days prior to collection. WS19T-CM from days 1, 3, and 5 day collections were applied to MDA-MB-231 cells at T0 of a wound closure assay and wounds closed for 16h. Each experiment was performed in triplicate and repeated thrice.
(TIF)

S2 Fig WS19T CAF CM differentially affects tumor cell motility. Monolayers of OVCA429, U251, MCF10A or MCF7 cells were wounded and were simultaneously incubated with either control DMEM or WS19T conditioned media for 16 h. Wound closure was measured in triplicate, and the experiment was repeated twice. *p<0.0001 relative to DMEM MCF7 controls.
(TIFF)

S3 Fig mDia2 localization in MDA-MB-231 cells is unchanged in response to CM. A, B. MDA-MB-231 cells plated on glass coverslips were treated with the indicated media for 8h before fixation. Cells were immunostained with anti-mDia2 antibodies, phalloidin and DAPI. Percent nuclear mDia2 fluorescence was measured relative to plasma membrane/cytoplasmic mDia2 fluorescent signal with Metamorph software. At least 30 cells per condition were measured and the experiment was repeated three times. Scale bars = 25μm.
(TIF)
