# Supplementary figures and images for: Carcinoma associated fibroblasts (CAFs) promote breast cancer motility by suppressing mammalian Diaphanous-related formin-2 (mDia2)
Source: PLoS One. 2018 Mar 29;13(3):e0195278. doi: 10.1371/journal.pone.0195278 (PMC5875872; doi:10.1371/journal.pone.0195278)

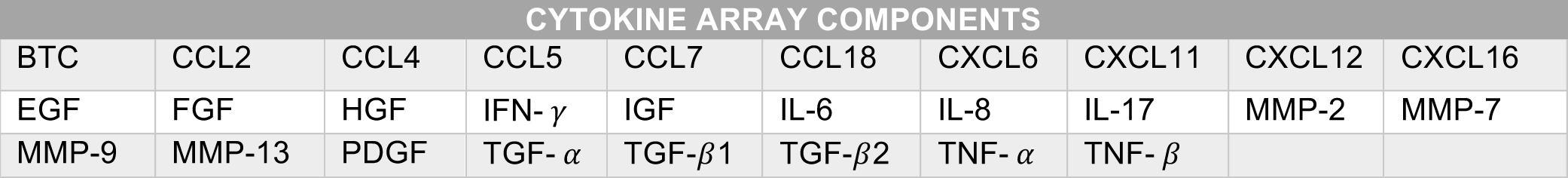

Supplement: S1 Table — (TIF) [file pone.0195278.s001.tif]

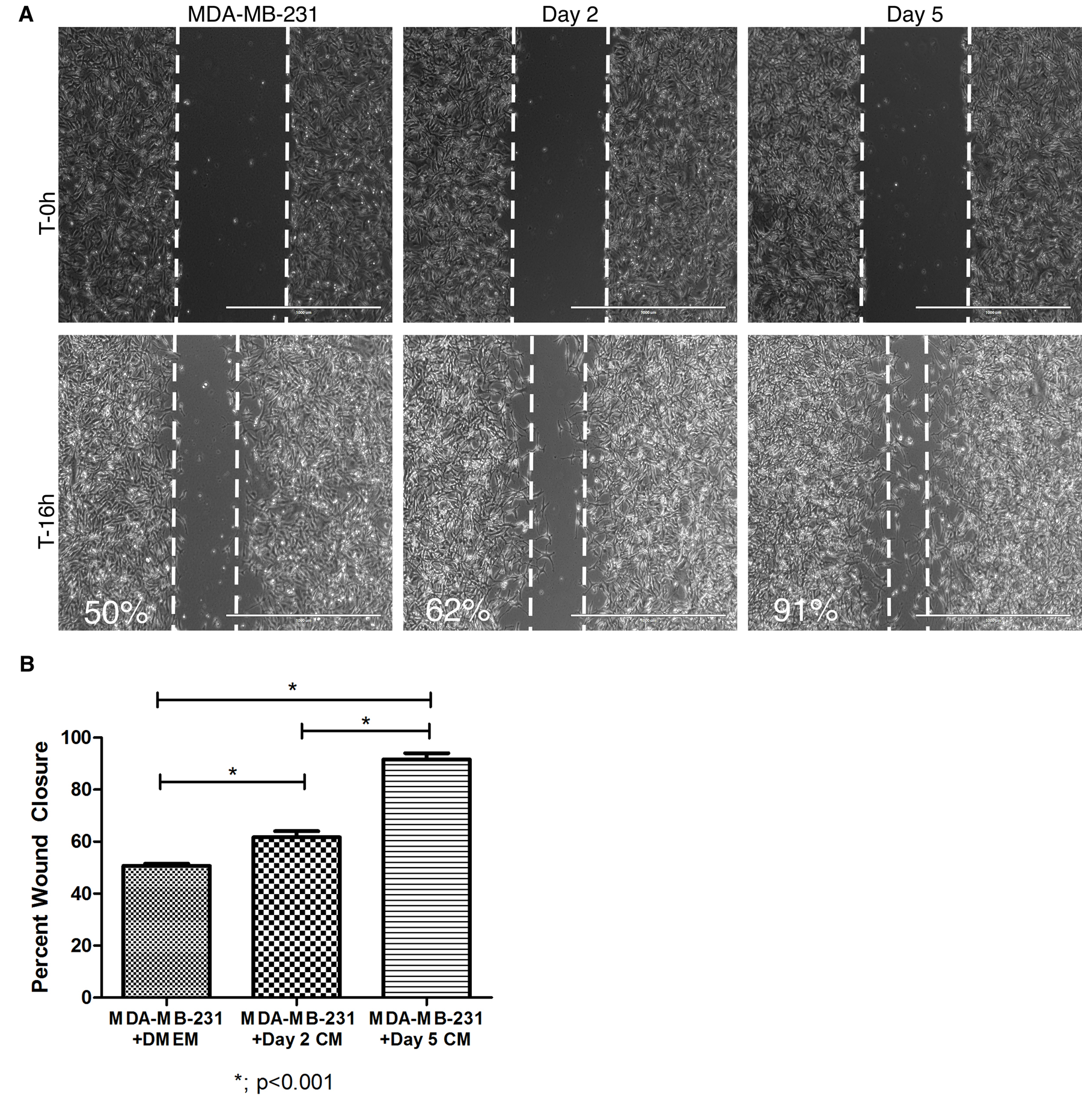

Supplement: S1 Fig — A, B. WS19T media was conditioned for 1–5 days prior to collection. WS19T-CM from days 1, 3, and 5 day collections were applied to MDA-MB-231 cells at T0 of a wound closure assay and wounds closed for 16h. Each experiment was performed in triplicate and repeated thrice. (TIF) [file pone.0195278.s003.tif]

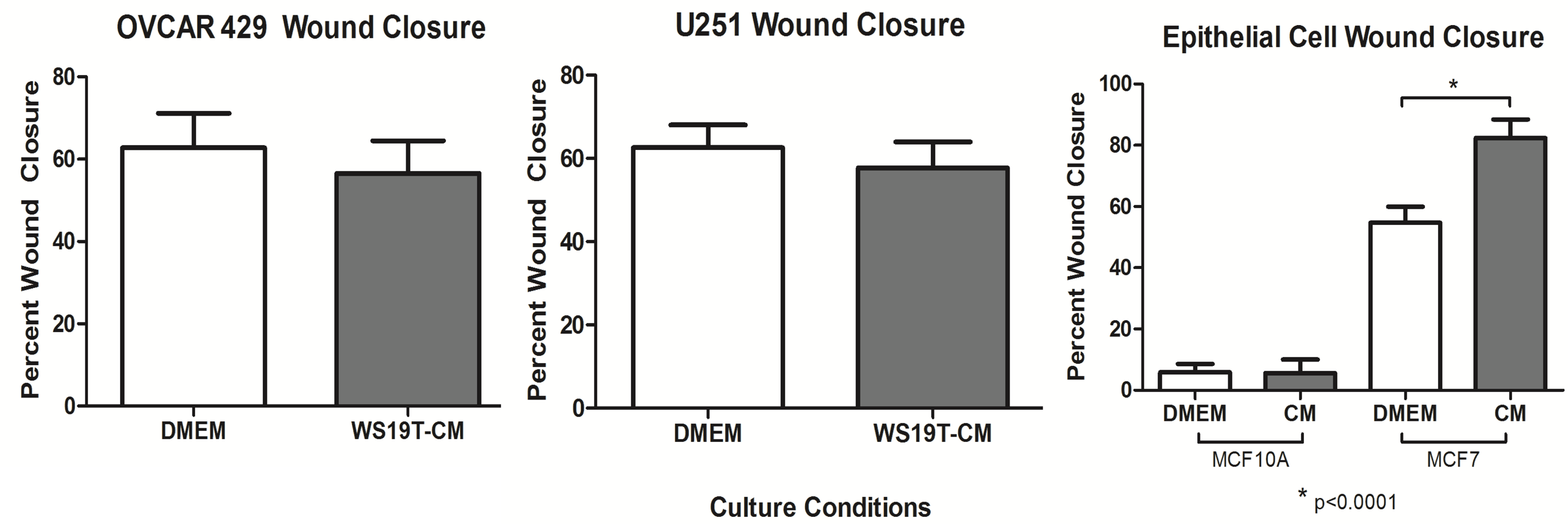

Supplement: S2 Fig — Monolayers of OVCA429, U251, MCF10A or MCF7 cells were wounded and were simultaneously incubated with either control DMEM or WS19T conditioned media for 16 h. Wound closure was measured in triplicate, and the experiment was repeated twice. *p<0.0001 relative to DMEM MCF7 controls. (TIFF) [file pone.0195278.s004.tiff]

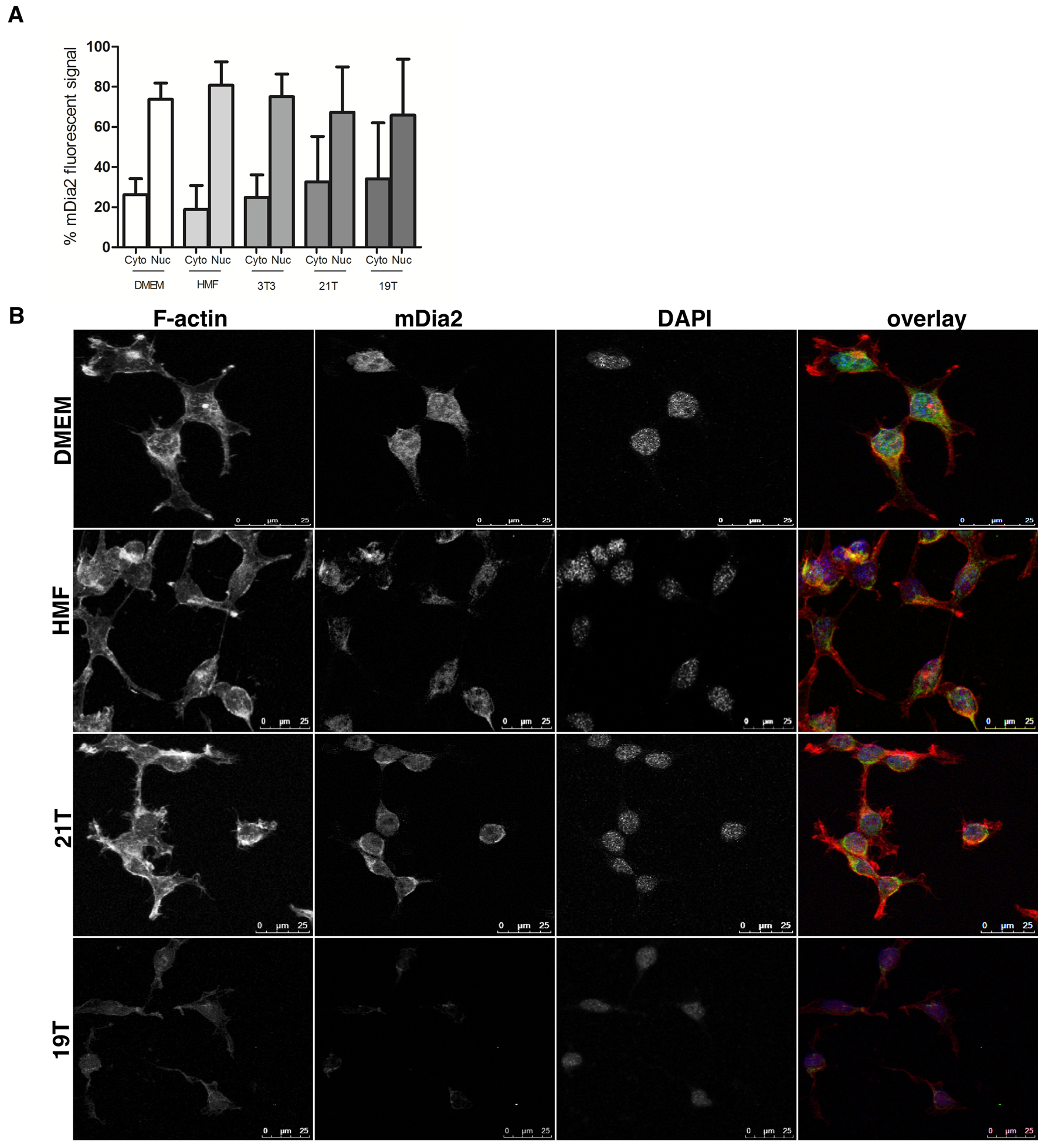

Supplement: S3 Fig — A, B. MDA-MB-231 cells plated on glass coverslips were treated with the indicated media for 8h before fixation. Cells were immunostained with anti-mDia2 antibodies, phalloidin and DAPI. Percent nuclear mDia2 fluorescence was measured relative to plasma membrane/cytoplasmic mDia2 fluorescent signal with Metamorph software. At least 30 cells per condition were measured and the experiment was repeated three times. Scale bars = 25μm. (TIF) [file pone.0195278.s005.tif]
